# Supplementary material for: Are carbon emissions trading and green financial instruments synergistic? -Comprehensive quantitative research based on content analysis
Source: PLoS One. 2024 Mar 7;19(3):e0298601. doi: 10.1371/journal.pone.0298601 (PMC10919601; doi:10.1371/journal.pone.0298601)
Supplement: S1 Data — (ZIP) [file pone.0298601.s001.zip › Data/地级市Z_F工作报告.zip!/╘╞─╧/▒ú╔╜╩╨/└┤╫╘╣1⁄2╓┌║┼í╛┬φ┐╦╩2╛▌═°í┐_2015.docx]

各位代表：

现在，我代表市人民政府向大会报告工作，请予审议，请市政协各位委员提意见。

一、2014年工作回顾

2014年是极不平凡的一年。在市委的坚强领导下，市人民政府认真贯彻落实党中央、国务院、省委省政府的决策部署，坚持稳中求进，努力克服困难，实现了经济平稳发展、社会和谐稳定。经预测，全市生产总值突破500亿元大关、达到501亿元、增长11%，规模以上固定资产投资增长28%，社会消费品零售总额增长13%，进出口总额增长29.3%，公共财政收入增长10.1%，城乡常住居民人均可支配收入分别增长11%和13.5%以上。城镇登记失业率、人口自然增长率、居民消费价格涨幅、单位生产总值能耗均控制在预期目标以内，多数指标增幅全省排位靠前。

（一）经济保持稳健增长

围绕发展目标，坚持壮大规模、打造基地、优化结构、促进增长，出台16项政策措施，加强分析监测，强化要素保障，严格督促检查，加大协调力度，确保了经济平稳运行。

农业农村健康发展。农业总产值和增加值分别达236亿元和138亿元，增长11.8%和6%。粮食生产实现“十一连增”，被列入全国24个产粮大市，省级名牌农产品发展到33个。收购烤烟132.46万担，在种植计划调减10.94万担的情况下，实现了产值、税收和烟农收入三不减；香料烟实现恢复性增长。传统种植产业稳步发展，特色优势产业势头强劲。绿化荒山成效明显，完成造林64.49万亩，发展核桃、油茶、糯橄榄共30万亩，实现林业总产值60亿元、增长13%。畜牧产业健康发展，产值达92亿元、增长8.8%。新型农业经营主体培育取得突破，被列入全国17个新型职业农民培育示范市，省级农业产业化龙头企业发展到43户，新建农民专业合作社208个、总数达1296个，消除“空壳村”123个。扶贫攻坚力度加大，争取到各类资金7亿多元，实施整乡推进项目4个，整村推进项目71个，解决了8万人的脱贫问题。

工业经济克难前行。预计第二产业增加值177亿元、增长15.4%。其中，工业增加值130亿元、增长15.1%；建筑业增加值47亿元、增长16%。规模企业获批入库35户、累计达172户，规模工业总产值、增加值分别达313亿元和106亿元、增长18.5%和14.6%。园区建设步伐加快，体制机制更加健全，配套举措更加全面，聚集效应更加显现，新增入园企业30户，完成工业增加值72亿元、增长20%。能源产业贡献突出，保山电力供电量增长11.9%，上缴税金增长45.6%，资产总额增长40%。培育小微企业，改革工商登记制度，民营企业和个体工商户达79750户、增长10.8%，从业人员22.95万人、增长9%，完成非公经济增加值233亿元、增长18%，占生产总值45%。全面完成节能降耗和淘汰落后产能目标任务，单位生产总值能耗下降1.8%。

第三产业稳中求新。围绕打造三张旅游名片，编制完成了珠宝之都和温泉之都总体规划，对旅游产业跨越发展进行了部署。火山热海国家5A级景区创建通过国家评审，蒲缥、永乐等5个温泉项目和杜鹃王、凯龙2个珠宝城项目建成投入运营。腾冲入选胡润榜2014全球优选生态旅游目的地，施甸怒江大峡谷旅游综合体规划建设呈现新亮点，善洲林场及松山大战遗址公园红色旅游、自驾自助旅游蓬勃发展。全年接待旅游总人数1050万人次、增长10.4%，总收入81亿元、增长23.6%。实施服务业改革，商贸服务业稳步发展，农村集贸市场建设成效明显，“万村千乡市场”网络基本形成，保山义乌国际商贸城一期市场建成运营。交通运输、批发零售等传统服务业稳步发展，仓储物流、电子商务等新兴服务业风生水起。启动国家信息惠民试点城市创建，引进浪潮集团等企业合作发展云服务，信息产业发展步伐加快，智慧城市建设取得阶段性成果。社会消费品零售总额达154.1亿元、增长13%，三产实现增加值186亿元、增长10%。

财税金融平稳运行。完成公共财政收入47.18亿元、增长10.1%，支出164.24亿元、增长9.3%。融资平台建设、农村普惠金融发展良好，融资工作成效显著，争取到国家开发银行棚户区改造贷款一期167亿元、二期49亿元。金融机构存贷款余额分别达650亿元和439亿元、增长17%和19%。

（二）投资工作成绩显著

完成规模以上固定资产投资369亿元、增长28%以上。其中产业投资150亿元、占40%，提升3个百分点。工业项目投资力度加大，建成标准厂房31万平方米，博翔弹性纤维、亿石达石材加工、福润肉类加工等一批项目建成投产，非电力工业预计完成投资65.4亿元、增长12.4%。水利建设投资20亿元，花园、红谷田等4座中型水库项目进展顺利，完成病险水库除险加固年度任务，新建成2.5万件“五小水利”工程，解决12万农村人口饮水安全问题。投入4亿元改造中低产田地30万亩。交通基础设施建设成效突出，完成投资40亿元，同比增长71%，大瑞铁路建设大保段加快、保瑞段开工，腾冲机场扩建工程完成前期工作和征地拆迁，龙瑞高速龙陵段、龙江特大桥、东绕城高速、昌宁大田坝至链子桥等公路建设进度加快，建成乡村通畅工程1290公里和250个招呼站。农村电网改造升级项目稳步推进，槟榔江、龙川江水电开发进度加快，施甸太阳能电站一期建成并网发电，能源产业投资29亿元、增1.5倍，电力装机容量达160万千瓦。完成服务业投资40亿元，其中旅游投资18亿元，松山大战遗址公园、施甸温泉古镇、昌宁小桥温泉等项目建设态势良好。

（三）城乡建设活力新现

城乡各类规划编制工作有序展开，省、市级特色小镇总体规划编制基本完成，村庄规划修改有序推进。中心城市建设步伐加快，人民路改造基本完成，永昌路改造及青堡路、保岫路、沙丙路建设正在抓紧推进，东城区“一湖四路”等项目建设步伐加快，城市综合体建设进展顺利。4个县城建设稳步展开，规划修编、功能配套、城市景观等重点项目建设齐头并进。抢抓国家棚户区改造的重大机遇，坚持把棚改与改善城市基础设施、提升城乡人居环境、完善住房保障体系、建设城市综合体有机结合，建成保障性住房1.5万套，特色城镇化的保山模式普获好评。立足长远、着眼百年，政府引导、市场主导，多元筹资、公司运作，投入12.6亿元建设34.88公里地下综合管廊，获得“把政绩埋在地下”的“良心工程”之点赞。“美丽保山”建设有序推进，腾冲入选“中国最美丽县”，昌宁被列“2014全国十大生态茶园县”。8个国家重点镇和37个省市级特色小镇建设进展顺利，和顺镇被评为第一批国家级美丽宜居小镇，列入全国传统村落94个、占全省的五分之一。整合资金10.05亿元，建设新农村试点611个，重点打造了腾冲银杏村和新歧村、施甸山邑村等一批美丽乡村。建设“一水两污”处理设施项目40个，建成农村生活垃圾焚烧炉130座，改造农村危房21440户。出台了个人住房公积金按月冲抵房贷等三项惠民政策，房地产业完成投资108亿元，商品房销售面积、销售额分别增长11.6%和16.6%。全市新增城镇建成区面积6.3平方公里，完成城镇基础设施建设投资46亿元、增长69%，城镇化率提高1.9个百分点、达40.5%。

（四）改革开放深入推进

政府职能转变和机构改革稳步推进，全面实施机构编制实名制管理，改革食品药品监督管理体制，整合组建市级卫生、计生机构，完成质监、工商系统接收移交。深入推进行政审批改革，市级审批项目由586项减为295项，初步实现投资项目集中审批“三级联动”。运用改革创新思维和市场化运作机制，“6995”服务平台、中心城市地下综合管廊建设和棚户区改造成为全国全省改革范例。有序推进沿边金融改革、“三农”金融服务、农村“三权抵押”等改革，林权抵押贷款余额达13.2亿元。龙陵及其他五乡镇农村承包土地确权登记颁证试点有序展开，隆阳、施甸探索农村土地承包经营权流转成效明显。财税、投融资体制和教育、文化、医药卫生及公共服务各个领域改革均取得较大进展。编制上报《昆—保—芒—瑞经济带保山建设规划》，成功举办“忠魂归国”等活动和第二届边交会，加强与缅甸克钦邦的交流与合作，密班公路前期工作取得实质性进展。提升口岸通关便利化水平，对外贸易增长较快，完成进出口总额2.6亿美元、增长29.3%。招商引资再创佳绩，引进项目383个、市外到位资金410亿元、增长30.1%。

（五）民生事业持续发展

统一城乡居民社会养老保险制度，参保人数达141.96万人。开发就业岗位2.2万个，城镇新增就业1.7万人。社会救助水平不断提高，城乡低保等困难群体保障标准提高15%以上，“一站式”即时结算医疗救助覆盖乡镇达90%。认真落实城乡居民收入“倍增计划”，城乡常住居民人均可支配收入增幅高于经济增幅，分别达21500元、7700元。全国质量强市示范城市创建全面展开，品牌创建、标准化建设和质量认证工作取得新成效，食品药品及农产品质量安全监管能力进一步提高。教育事业健康发展，“两基”水平巩固提升，校安校改工程进展良好，高中阶段教育毛入学率提高8个百分点，高考上线率连续8年高于全省平均水平。科技创新体系建设取得突破，国家级高新技术企业由7家增加到15家，腾冲制药厂入选科技部火炬计划重点高新技术企业，腾冲经开区获批省级高新技术园区，建立院士和专家工作站各1个，获国家和省科技经费3408万元、增长83%。滇西抗战纪念馆、国殇墓园和龙陵抗战纪念馆被国务院命名为全国第一批抗战纪念设施。实施国家公共文化服务体系示范区后续建设，保山历史名人堂建成开放，在全省率先实施数字图书馆推广工程，可移动文物普查和文化市场监管力度加大。与上海等发达地区医疗服务合作深入开展，卫生信息平台建设、中医药先进市创建有效推进，建立居民电子健康档案，妇幼健康计划等惠民工程顺利实施。公立医院综合改革、县乡医疗服务一体化管理试点进展良好，基本公共卫生服务均等化水平稳步提高，全市新农合参合率达98.95%。全民健身运动和竞技体育协调发展，残疾人文化体育示范市创建依计展开，成功举办云南围棋棋王赛，在亚洲残运会和省运会、民运会、残运会、七彩云南全民健身运动会上取得了好成绩；探索体育旅游，尝试举办芒宽怒江漂流活动取得成功。认真贯彻民族宗教政策，深入推进民族团结进步边疆繁荣稳定示范区创建，全面促进民族团结宗教和谐，少数民族地区发展加快。双拥工作取得新成绩，军民团结日益巩固，军政、军地关系更加密切。继续加强防灾减灾体系建设，公共突发事件应急机制不断完善。第三次经济普查取得阶段性成果。外事、侨务、史志、档案、人防、水文、气象、老龄及残疾人等工作健康发展。全市民生类支出125.5亿元，占公共财政预算支出的76.4%。

（六）社会治理再树典型

切实加强民主法制建设，“平安保山”建设成效显著。不断强化基层工作，落实维护稳定责任，完善社会治安防控体系，“6995”服务、“十户联防”治理实现城乡全覆盖，创新网格化服务管理模式、利用信息平台打通联系服务群众“最后一公里”的创举全省全国影响甚广。成功破获腾冲“1?30”特大持枪杀人案，新一轮禁毒人民战争取得显著成效，群众安全感满意率和执法队伍满意度测评分列全省第2名和第1名。“六五”普法正常展开，龙陵黄龙玉资源获得立法保护。强化安全生产责任，未发生重特大安全事故。深入落实群众工作六项制度，“一教育五行动”扎实推进，信访工作成效明显，有效解决茄子山水库移民等历史遗留问题。大力弘扬善洲精神，认真践行社会主义核心价值观，评选表彰了36名市级劳模。创新社会治理的“保山经验”成为“2014全国社会治理创新案例”20个优秀案例之一，和顺镇获“首届中国治理创新100佳经验”中的“文化发展10佳经验”奖。

（七）政府建设成效明显

认真学习贯彻习近平总书记系列重要讲话精神，扎实开展党的群众路线教育实践活动，坚决贯彻中央“八项规定”，加强作风建设与破解发展难题相结合狠抓整改落实，正风肃纪渐成常态，“四风”得到根本扼制，工作作风大为转变，政治纪律性明显增强。全面清理超标办公室和公务用车，市政府会议数、文件数、“三公”经费支出分别下降18.2%、21%、33.7%。大力推行政务公开，政务服务体系进一步完善。廉政责任得到严格落实，行政监察和审计监督进一步强化，行政成本控制全面加强，公务出差、公务接待等进一步规范。依法接受市人大及其常委会的法律监督、工作监督和市政协的民主监督，自觉向市人大及其常委会报告工作，向市政协通报情况，认真听取各民主党派、工商联和无党派人士的意见建议，积极支持工会、共青团、妇联等群团组织发挥桥梁纽带作用，主动接受人民群众、新闻媒体和社会各界监督。全年共办理人大代表建议51件、政协提案195件，办复率、面商率均为100%，大多数取得良好效果、获得好评。

各位代表，成绩令人振奋，这是坚决贯彻中央精神的结果，是省委、省政府正确领导、关心帮助的结果，是市委统揽全局、协调各方、科学决策、综合施策的结果，是市人大、市政协有效监督和有力支持的结果，是全市各族人民团结奋斗、攻坚克难、共同努力的结果。在此，我代表市人民政府，向全市各族人民，向人大代表、政协委员，向各民主党派、工商联、无党派人士和各人民团体，向驻保解放军指战员、武警部队官兵和公安政法干警，向离退休老同志，向在我市创业的投资者、建设者，向所有关心支持保山发展的同志们、朋友们致以深深敬意和衷心感谢！

认真反思，我们必须看到，去年工作中还存在诸多问题，全市生产总值、财政收入、城乡常住居民人均可支配收入等没有完成年初预定目标。这既有经济下行压力加大、国家税收政策调整等客观原因，也有对经济规律把握不够准确、采取应对举措不够及时、点对点破解难题不够有力、工作措施落实不够到位等主观方面的问题。对此，我们必须认真反省检审，切实加强整改，争取在新的一年创造新的业绩。

二、2015年目标任务

各位代表，今年是全面深化改革的关键之年，是“十二五”的收官之年，也是规划“十三五”的谋划之年。挑战与机遇并存，困难与优势同在。世界经济仍将延续缓慢复苏之势，国内经济转入新常态，云南发展进入新阶段。从保山实际看，产业发展不配套、人才支撑不足、交通基础设施滞后尤其是境外通道不畅的瓶颈制约，主要工矿产品价格下滑，财政和城乡居民持续增收难度加大等，诸多因素可能使我们面临更多更大的困难。但是，国家“一带一路一廊”战略的实施，给保山带来了重大的发展机遇。省委、省政府大气魄打造云南经济升级版，着力编织交通、航空、水保障、能源、信息五大网络，有利于保山加快基础设施建设和产业转型升级发展。经过多年不懈努力，“四化”进程持续推进，我市发展条件不断改善，广大干部群众干事创业精神更加饱满，为保山实现更好更快发展积蓄了强大的正能量。我们要科学认识新常态，准确把握新常态。与沿海发达地区长时期高速发展相比，保山虽然近几年获得了高速发展，但发展还很不充分，保山经济发展的强烈韧性和巨大潜力尚待释放，产业布局好、开放空间广、发展动力足、增长余地大。我们必须立足保山发展的阶段性、区域性特征，深刻认识在有质量有效益的前提下，中央鼓励有条件的地方发展得快一点，保山加快发展符合全国区域协调发展的要求，保山必须提速发展才能完成好省委省政府赋予的使命和责任，确保与全国同步全面建成小康社会。我们必须坚决摒弃把新常态看作就是增速“慢下来”、就是“弱化发展”、就是任其“自然增长”等片面认识，切实增强使命意识、机遇意识、创新意识、担当意识、赶超意识，坚持以经济建设为中心不动摇，坚持以加快发展为第一要务，坚持发展规模、速度、质量、效益的有机统一，坚定发展信心，切实做到稳中有进积极进、稳中有为主动为，推动经济转型升级提质增效，创造新常态下加快发展的保山模式。

根据党的十八大、十八届三中、四中全会、中央经济工作会议和省委九届九次全会、省委经济工作会议精神，按照市委三届五次全会的决策部署，今年政府工作的总体要求是：高举中国特色社会主义伟大旗帜，以邓小平理论、“三个代表”重要思想、科学发展观为指导，深入贯彻落实习近平总书记系列重要讲话和对云南工作的重要指示精神，坚持稳中求进总基调，坚持规模与质量、速度与效益相统一，准确把握经济发展新常态，抢抓“一带一路”建设重大机遇，着力深化改革、扩大开放，全面推进依法行政，一以贯之地实施“六大战略”和“四化五加强”举措，一鼓作气地做大做强骨干产业，竭力打造旅游名片，加快培育文化品牌，更加突出固定资产投资和基础设施建设，更加突出民生改善和和谐社会建设，更加突出环境保护和生态文明建设，全面实现“十二五”规划目标任务，争当全省经济转型升级发展排头兵。

2015年经济社会发展主要指标建议为：生产总值增长10%，规模以上固定资产投资增长20%，社会消费品零售总额增长12%，进出口总额增长15%，公共财政预算收入增长8%，金融机构存贷款余额均增长14%，城镇、农村常住居民人均可支配收入分别增长10%和12%，城镇登记失业率控制在4.3%以内，居民消费价格涨幅控制在3%以内，单位生产总值能耗下降1.6%。

围绕上述目标，必须从以下五大方面着力：

（一）坚持转方式调结构，同心同德做大做强产业

突出增收抓三农。做大基地，做强龙头，做优品牌，培育主体，强化投入，加快农业产业由资源型向商品型、品牌型转变，大力发展产出高效、产品安全、资源节约、环境友好的现代农业。实现农业增加值147亿元、增长6%以上。

大力推进农业产业化。聚力发展高原粮仓、特色经作、山地牧业、高效林业、淡水养鱼和开放农业。稳步提升粮油基础产业，实施永久农田划定和设施农田地保护，严守耕地保护“红线”和播种面积底线，认真落实粮食行政首长负责制，用好全国“产粮大市”扶持政策，确保实现粮食产量143万吨、增1.4万吨。加快发展畜牧业，完成产值98亿元、增长6.5%。认真落实“两烟”科技政策措施，调整结构、提质增效，确保全面完成任务。着力提高核桃、茶叶、咖啡、蚕桑、油茶等产业的质量和效益。建立风险防范机制，大力发展冬早蔬菜、澳洲坚果、草果等高原特色优势产业，培育壮大石斛、中药材、食用菌、糯橄榄等新兴产业，探索发展光伏农业，争取上级“光农互补”项目资金支持，研究实施“光伏食用菌”强村惠农工程。抓紧规划建设隆阳区国家级现代农业示范区、腾冲界头生态农业园区、潞江坝生态文化产业园、国家绿色生态优质烟叶基地，推进农业产业基地和粮食生产功能区建设，重点打造一批生态农业专业村。加快建设隆阳、腾冲、昌宁生物资源加工和商贸园区，塑造生态农业的保山品牌。

加快培育新型农业经营主体。着力培育农业重点龙头企业，争取新增销售收入上亿元企业5户、省级龙头企业6户、市级龙头企业20户。大力发展农民专业合作社，培育新型职业农民和专业大户，发展种粮大户60户以上，新增工商注册登记的家庭农场20户以上，培育省级农民合作示范社8户以上。消除“空壳村”110个，加快农村集体经济的发展。

突出园区抓工业。以加快园区建设为重点，推动产业一体化、集群化发展，实现工业增加值145亿元、增长13%，单位生产总值能耗下降1.6%，全面完成节能减排任务。

完善工业园区产业发展规划，突出优势错位发展，加快培育生物资源加工、能源、信息、轻纺服装等产业，大力提升冶金矿产和建材产业。继续加强招商引资，年内新增入园企业30户以上。全力推进工贸园区轻纺产业园、信息产业园、木材加工交易园，腾冲综合物流产业园，水长园区石材产业园等专业园区建设，抓实抓牢重点项目，加快构建银行、园区、企业融资担保合作机制。力争5个省级工业园区实现工业总产值260亿元、增长21%。

大力发展民营经济，认真落实中小企业、民营经济健康发展的各项政策，抓好保山工贸园区孵化器建设，努力为小微企业、创业企业入园发展创造条件，加快“两个10万元”微型企业培育，建立企业减负长效机制，帮助民营企业提高经营管理水平。确保民营经济增加值占全市生产总值47%以上。

切实帮助企业解决“融资难、用工难”等问题，健全丰水期硅电价格联动机制，强化生产要素保障，确保企业正常生产。抓好企业育规工作，争取年内新增规上企业24户。

突出服务抓三产。围绕旅游国际化和建设区域性国际商贸物流基地目标，加快旅游产业转型升级，积极发展现代服务业，实现第三产业增加值210亿元、增长10%。

加快发展旅游产业。全面打造“三张旅游名片”，大力发展康体旅游、休闲旅游、乡村旅游，全力推动旅游业由观光型向康体度假、休闲购物转型发展，加快形成“龙头带动、两翼驱动、三都联动、全面推动”的新格局。编制温泉国家公园建设规划，规划建设一批温泉度假区、温泉旅游小镇，多层次开发温泉产品，积极发展养生养老、美容美食等配套产业，着力打造温泉之都；加快中心城市珠宝交易中心建设，提升翡翠、黄龙玉、南红玛瑙加工交易水平，强化市场诚信体系建设和规范化管理，着力打造珠宝之都；加强特色旅游小镇建设，强化旅游要素配套，加快高黎贡山国家公园建设，积极支持施甸怒江大峡谷旅游综合体、腾冲雅居乐旅游小镇二期工程、龙陵龙川江生态文化康体休闲旅游度假区、昌宁10万亩樱花园等项目的规划建设，着力打造休闲之都。成立旅游投资公司，加强优质旅游资源的引导性开发。加大旅游宣传促销力度，增强保山旅游知名度和影响力。加强市内外旅游环线规划建设，增强旅游业联动发展能力。大力发展智慧旅游、航空旅游，增开省外直航班机，降低旅游成本，加强行业管理，提升服务质量。确保旅游总人数、总收入分别增长10%和20%。

促进服务业全面发展。认真落实促进消费政策措施，增强社会消费能力。稳步推进服务业发展试点改革，加快商贸物流业向大商贸、大物流发展，完善城乡流通体系，积极推进保山义乌国际商贸城二期工程等项目建设和招商拓展，加强物流基础设施建设。扎实推进信息惠民国家试点城市创建，加快信息产业园和灾备中心等项目建设，大力发展云服务、电子商务、冷链物流和快递物流等产业，加快云计算平台建设，着力建设智慧保山。努力扩大文化娱乐、体育健身等服务性消费。实现社会消费品零售总额173亿元、增长12％。

（二）坚持抓当前谋长远，同心同德增强活力动力

科学谋划“十三五”。加强发展趋势预判，遵循我国经济发展大逻辑，服务国家区域发展大战略，服从云南转型发展大格局，把握保山加快发展大趋势，立足新起点、确立新理念、制定新目标、谋划新举措、构建新常态，扎实抓好重大课题研究、总体规划和专项规划编制工作，科学绘制未来5年发展宏伟蓝图，确保与全国全省同步实现全面建成小康社会目标。

持续强化投资拉动。着力构建综合交通、能源、水利和信息网络，实施一批基础设施、产业和民生项目，力保投资对经济增长的强力拉动。确保完成投资436亿元、增长20%以上。

不断完善投资工作责任制、项目建设督查制、重大项目挂钩制，真正落实项目牵头挂钩人的责任，切实解决项目建设中的困难和问题。交通方面，积极推进两个机场改造、大瑞铁路、龙江特大桥、龙瑞高速、保怒高速等重点项目建设，加快东绕城高速、大链公路等国省干线建设，争取隆阳至施甸公路改造、黄草坝至南伞二级公路开工建设，组织实施好重要县道改造、1333公里建制村通畅工程及350个客运招呼站建设，加速天猴高速昌保段、怒江流域航运等项目前期工作。能源方面，加快农网改造升级、天然气综合利用，加强电源电网建设，抓好槟榔江、龙川江龙头水库及电站等水电建设项目。工业方面，加强腾冲玄武岩纤维产业园、龙陵金属硅产业链延伸等项目前期工作，着力推进复烤厂迁建，积极协调中柏韩美服装、宝佳鞋业等项目开工建设。加紧力元祥石业、摩尔农庄等项目建设，力争年内建成投产。农业农村方面，抓好以农田水利、能源、生态环保等为重点的基础设施建设，加快推进花园、红谷田、段家坝等在建中型水库及保山坝西水东调、龙陵勐糯东山引水工程等重点水利项目建设步伐，开工建设腾冲小地方、昌宁松林等水库，改造中低产田地25万亩，治理水土流失110平方公里，新增有效灌溉面积4.5万亩，解决9.3万人的农村饮水安全及缺水问题。旅游产业方面，推进高黎贡山旅游度假区规划建设，抓好腾冲10个旅游小镇、松山大战遗址公园、施甸温泉养生洗浴及昌宁小桥温泉等一批项目建设。

强化项目前期工作，加强项目策划，扩充完善项目储备，大力推行集中审批、并联审批，完善项目限时办结制。改善项目落地服务，加快土地、环评、林评等工作进度。加强向上汇报争取，力争一批重大投资项目获得国家和省的支持。

切实抓好财税金融。强化财税管理，扩大融资规模，保持财政金融稳定运行，增强经济社会发展保障能力。

严格财政管理。深入推进财税体制改革，认真落实新预算法，严格预算约束，将政府收入和支出全部纳入预算管理，全面推进预算公开，进一步完善财政转移支付制度。充分发挥财税政策和财政资金的引导作用，吸引社会资金参与经济建设，重点支持园区发展、骨干产业和重点项目建设，积极培植新兴财源。抓好重点税种、重点行业、重点企业税收征管，健全税收征管体系和非税收入收缴体系，确保完成公共财政预算收入50.96亿元、增长8%。优化公共财政支出结构，重点保障民生建设、机构运转和社会稳定，严格控制一般性支出，切实降低行政成本，确保“三公”经费零增长。进一步强化财政监管和审计监督，严肃财经纪律，加强政府债务管理，不断提高财政资金使用效益。

强化金融支持。加快推进沿边金融综合改革试验区建设，努力构建多层次的金融服务体系，鼓励银行和保险机构延伸服务网点，不断扩大金融覆盖面。突出园区重点，加大中小微企业支持力度，提高金融服务实体经济的能力。加强农村信用和支付体系建设，继续推进“一创两建”，扩大农业保险品种和规模，提高农村金融服务水平。探索建立以存贷比为主要指标的信贷激励机制，加强重点建设项目与金融机构的协调，着力搭建政、银、保、企合作平台，进一步扩大社会融资规模。认真筹划市国资公司第三期发债工作，鼓励符合条件的企业通过债券融资等方式筹集资金。规范发展民间金融和融资性担保公司，积极探索金融创新，引导和支持中小企业到新三板挂牌。加快推进社会信用体系建设，加强金融行业自律和监管，严格管控金融风险。确保金融机构存贷款余额分别达741亿元和500亿元、增长14%。

大力推进改革开放。坚持以改革统领全局，加快发展方式创新，全力营造保山大开放、大招商、大发展的新格局。

继续推进政府职能转变、行政审批和机构改革，加大政府购买服务力度，探索资源性产品价格形成机制、公共资源市场交易机制和生态补偿机制改革，完成公务用车制度改革任务。加快推进以人民币跨境结算和跨境使用、小币种兑换等为重点的沿边金融开放和跨境金融改革创新，促进贸易投资便利化。稳步推进农村综合改革及农业、林业、水利、供销社等专项改革，进一步释放“三农”发展活力。全面推进农村“三权”确权登记颁证工作，进一步探索实施“三权”抵押担保融资、农村土地承包经营权有序流转和多种形式适度规模经营。加快公墓建设，推进殡葬改革。推进投融资、财税、户籍等重点领域改革。

继续深化与长三角、珠三角等重点地区经济、文化、人才合作，巩固两个机场现有航线，争取开通保山至北京和杭州、腾冲至上海和广州4条航线，恢复腾冲至大理航线，力争开通境外航线。积极推动昆—保—芒—瑞经济带建设，加快建设黑泥塘边民互市区，争取猴桥边合区获批国家级边合区。加快推进国际大通道建设，确保密支那至班哨二级公路开工建设，争取保腾铁路、腾猴高速等项目前期工作取得重大进展，着力推进与周边国家互联互通。加强与缅、印、孟等国的交流合作，充分发挥海外华人华侨作用，鼓励企业和民间组织开展对外贸易、旅游、投资、文化等合作，全力争取在孟中印缅经济走廊建设中率先突破，着力打造沿边开放新高地。加大出口企业扶持力度，加强对重点行业、重点企业、重点产品的跟踪服务，进一步提高通关便利化水平，实现进出口总额3亿美元、增长15%。

继续加强领导带头招商、部门招商、产业招商、以商招商，努力形成招商引资强大合力。强化项目储备、策划、包装、推介，全面推行项目全程服务，力争在大数据、轻纺、五金和健康等产业上，引进一批技术水平高、带动能力强、产业延伸性好的大项目。引进市外资金470亿元、增长15%。

（三）坚持绿色发展理念，同心同德建设美丽保山

全面推动绿色发展。围绕争当全国全省生态文明建设排头兵的目标，加快实施《保山市生态市建设规划》，启动全国生态文明示范市创建工程。进一步优化国土开发空间布局和城乡产业布局，抓实中心城区工业企业入园工作，推动工业向园区集中，城市向生态、低碳、环保转型。加快经济结构调整和发展方式转变，加速生态优势向经济优势转化，大力发展绿色种养植及其加工、清洁能源、绿色建材、生物制药、旅游文化、商贸物流、信息技术等绿色产业，繁荣绿色文化，倡导绿色消费、绿色出行，建设绿色家园、绿色走廊。引导企业引进节能新产品、新技术，全面推进重点领域节能，深入开展全民节能行动。加快“森林保山”建设，抓好人工造林、低效林改造和森林火灾植被恢复，落实11.3万亩绿化荒山行动，实施退耕还林2.16万亩。实施好国家新一轮退耕还林还草和云南陡坡地治理工程，努力建设生态安全屏障。

强化资源环境监管。实施最严格的耕地保护、节约用地和水资源管理制度，推进矿产资源采、选、冶一体化发展，不断提高资源利用效益。强化森林资源和野生动植物保护管理，全力抓好森林防火。深入贯彻落实新环保法，加强环境监测预警和综合执法，加速淘汰落后产能，落实节能减排措施，依法督促重点行业、企业提高污染治理水平，切实降低噪声、大气、土壤、水体污染，继续建设农村垃圾处理设施，稳步提高城镇生活垃圾无害化处理率、工业固体废物处置利用率，不断提升环境质量。

规划建设绿色城乡。围绕建设美丽家园，加强城市规划管理，着力建设宜居宜游宜业的生态城市。抓好城市总规和专项规划的有机衔接，完善集镇规划、村庄规划，建立规划管理信息平台，积极推行“阳光规划”，提高规划管理水平。

推进生态城镇建设。坚持“双城联动、产城融合”的发展思路建设中心城市，加快完善北城区、东城区、青阳片区基础设施，扎实推进“一湖四路”、“三校一院”、地下综合管廊和城市综合体等重点项目建设，完成保岫路建设、永昌路改造。加强中心城市市场规划建设，采取有力措施加大闲置土地清理力度，推进合理开发利用。全力打造腾冲国际知名旅游目的地，大力推进龙陵“山水玉”特色旅游城市、施甸新兴旅游城市和昌宁山水田园城市建设。促进4个县城地下综合管廊建设，继续加大城镇公用设施建设和园林绿化建设投入，建设美丽宜居新型城镇。加强集镇建设，集中力量建设10个省级、27个市级特色小镇，深入抓好姚关、勐糯、柯街、卡斯等城乡统筹发展试点工作。认真落实农业转移人口各项权益保障措施，有序推进农业转移人口市民化。推广中心城市打破行政区域集中安置的经验，加大土地收储和城市经营力度，多种形式盘活城市资源和资产，强化产业配套，加快城市产业和商贸发展，积极促进房地产业健康发展。实现建筑业增加值54亿元、增长13%，城镇化率达到42%。

加快美丽乡村建设。以中心村、特色村和传统村落为重点，完善村庄规划，开展人居环境提升行动，建设各类试点村500个，打造美丽乡村示范点40个。大力发展乡村生态旅游，加强传统村落、民居和非物质文化遗产的保护利用。加强农村环境的管理与维护，健全村庄基础设施、公共服务设施管理和环境卫生保洁机制，实施农村环境综合整治行动。

提升城市管理水平。强化综合执法，推进网格化管理和平安社区创建。加强小街小巷、建筑工地、城郊结合部等市容薄弱环节整顿和管理，专项整治乱停乱放、私搭乱建、噪声污染、建筑扬尘，扎实开展中心城市公共交通和环境卫生综合整治，提升城市公交服务能力和环境卫生质量，不断完善城市公共服务体系。

（四）坚持惠民生保稳定，同心同德促进社会和谐繁荣

统筹发展社会民生。以保障和改善民生为天职，更加注重保障基本民生，更加关心低收入群众生活，更加关注发展社会事业，更加重视社会和谐稳定。

认真落实就业创业政策，积极发展劳动密集型产业、现代服务业和中小微企业，开发就业岗位2.65万个，新增就业1.65万人。发展产业促进就业，支持创业带动就业，加强职业技能培训，抓好就近就地就业，切实解决轻纺服装、电子信息等产业发展的用工需求，重点做好高校毕业生、复转军人、农转城人员、下岗失业人员、城镇就业困难人员等重点人群的就业、创业培训指导工作。切实维护劳动者合法权益，着力构建和谐劳动关系。

努力实现城乡居民社会养老保险制度全覆盖，继续完善职工养老、城镇医疗、失业、工伤、生育保险制度。强化社会保险稽核，加大社会保险费征缴和清欠力度，确保各项待遇按时足额发放。扎实抓好棚户区改造、保障性安居工程建设，建设保障性住房9030套（户），有效解决城镇低收入群体、新就业人员、农民工的住房困难。逐步建立新型社会福利体系，完善社会救助制度，坚持托底线、救急难、可持续的原则，统筹抓好最低生活保障和各种救助，切实保障公民基本生活，维护社会和谐稳定。大力发展老龄服务事业和养老产业，加强社区养老服务设施建设，推进城乡特困老人服务体系建设，健全残疾人社会保障和服务体系。

扎实推进连片扶贫和精准扶贫，大力推进整乡整村开发，投入扶贫资金7亿元以上，力争贫困人口减少8万人。

深化收入分配制度改革，落实国家强农惠农富农政策，推进基本公共服务均等化，千方百计增加居民收入。着力加强中心城区农贸市场建设，改进市场监管，努力平抑物价，切实保障消费者权益，营造放心消费环境。健全食品药品安全监管体系，加强全程监控，保障人民群众饮食用药安全。着力强化品牌培育，深入推进“全国质量强市示范城市”创建活动。

全面巩固“普九”成果，促进教育资源优化配置，推动义务教育均衡发展。扎实抓好高中教育“扩容提质”，加快发展职业教育，力争高中阶段毛入学率达到85%以上。积极支持保山学院迎评促建及其华文学院、“国门大学”和食品安全研究院保山分院建设，抓好中医药高等专科学校改革发展，加快滇西应用技术大学珠宝学院建设，确保招生办学，规划筹建轻纺服装、生物制药、信息技术、环境农业等学院，促进高等教育加快发展。全面落实“两免一补”和营养改善补助政策，解决好进城农民子女接受义务教育问题。抓好薄弱学校改造等工程，加快职教园区建设，不断改善办学条件。积极扶持民族教育、民办教育、学前教育、成人教育发展，办好各级老年大学，推动教育事业健康发展。

加强科技创新平台建设，狠抓科研成果转化和知识产权保护，加强先进实用技术培训和推广，加强科技项目申报和人才队伍建设，不断改善创新环境，激发科技创新活力，着力提升创新能力。

围绕打造“五大文化品牌”，抓实国家公共文化服务体系示范区后续建设工作，抓紧编制文化品牌建设规划，推进文化与旅游融合发展，加快文化产业发展步伐，提升公共文化服务水平。继续深化文化体制改革，加强文化市场监管，构建现代公共文化服务体系和现代文化市场体系。加大文化设施建设投入，充实乡村文化站馆内容，倡导“全民阅读”计划，切实加强城乡文化建设。

完善新农合和基本药物制度，加快推进城乡居民大病保险试点工作。继续加强基层医疗卫生服务体系建设，支持民营医疗机构发展，完善城乡医疗卫生服务网络，积极创建全国中医药工作先进市。加快推进卫生信息化，提高科学化管理水平，加强与上海等发达地区合作，抓好人才培养，争取实现各县区均有1个以上省级临床重点专科，努力争创全省一流医疗卫生水平，打造区域性医疗养老服务中心。积极推进光明工程、妇幼健康计划等公共卫生服务项目，抓好重大传染病防治和突发公共事件医疗卫生救援工作，依法加强精神卫生工作。完善县乡村人口和计划生育服务网络，抓好流动人口计划生育管理，促进人口均衡发展。

认真落实全民健身国家战略，关心老年人健康事业，加强青少年体育工作，广泛开展全民健身运动。进一步发挥体育场馆功能作用，研究解决奥新体育馆开放利用问题，抓好永子棋院等文化体育建设项目，积极争取承办国际国内围棋赛事，筹备好第四届市运会。深入贯彻落实党的民族宗教政策，抓实民族团结进步边疆繁荣稳定示范区“555”示范点创建工作，促进民族地区加快发展、宗教领域和谐稳定。加强全民国防教育和动员，继续抓好双拥工作，进一步密切军民、军政、军地关系。实施好妇女儿童发展规划纲要，切实维护妇女儿童合法权益。继续抓好外事、史志、档案、人防、老龄、残疾人等工作，促进各项事业全面发展。

构建核心价值体系。深入学习善洲精神，弘扬社会主义核心价值观，着力构建社会主义核心价值体系，深入开展“一教育五行动”和社会公德、职业道德、家庭美德、个人品德教育，倡导德行善举、见义勇为，扎实推进志愿者服务、俭约保山等活动，着力提高公民文明素质，努力建树社会文明新风尚。

促进社会和谐稳定。加强和改进信访工作，大力推行联合接访和视频接访，逐步推行律师参与信访工作制度，建立涉法涉诉信访依法终结制度。推进社会治安防控体系建设，依法打击各类违法犯罪活动，深入开展禁毒防艾人民战争。强化边境管控，维护边疆安宁。全面落实安全生产党政同责、一岗双责，加大源头排查治理，严防重特大事故，切实保障人民群众生命财产安全。抓好防灾减灾工作，完善气象、地质、地震等监测预报预警，提升应急救援能力。加强平安社区建设，完善“6995”网格化管理，培育社会中介组织，全面提升基层民主管理水平。强化矛盾源头化解和突发事件应急管理，推行预防化解重大矛盾纠纷包保责任制，深入落实群众工作六项制度，提升社会治理法治化水平。切实维护征地拆迁中的群众利益，做好棚户区改造等工程移民安置工作，完善和创新流动人口、特殊人群服务管理，重视农村留守儿童、妇女、老年人问题，创新基层治理，加强稳定风险评估，健全舆情监测、分析、引导和处置机制，确保社会和谐稳定。

（五）坚持加强依法行政，同心同德建设法治政府

全面推进依法治市。深入贯彻落实全面推进依法治市实施意见，统筹推进法治保山、法治政府、法治社会建设，为改革开放发展提供有力的法治保障。做好地方立法准备，积极稳妥推进城市建设管理、环境资源保护等领域立法，配合支持昌宁田园风光立法保护。着力建设法治社会，抓实法治教育，落实普法责任，加大法治创建力度，使学法尊法守法用法成为全市人民的共同追求。加强法治队伍建设，不断增强法治人才保障。大力促进公正司法，努力让人民群众在每一个案件中都感受到公平正义。

加快建设法治政府。依法推进政府职能转变，提高政府执行能力和治理水平，加快建设职能科学、权责法定、执法严明、公平公正、廉洁高效、守法诚信的法治政府。抓好行政审批项目接、管、放工作，推行权力清单、责任清单和负面清单制度，推进政府机构、职能、权限、程序、责任法定化，促进政府管理从事前审批向事中事后监管转变。推行政府法律顾问制度，严格执行规范性文件备案审查制度，推进行政复议规范化，健全政府依法决策机制，建立健全重大决策目录管理、社会风险评估、合法性审查、群众参与、终身责任追究和责任倒查6个机制，探索建立第三方评估论证制度，确保重大决策于法有据、程序严格、过程公开、责任明确。努力构建科学有效的权力运行制约和监督体系，建立领导班子分析研判制度，增强督查合力和实效。深化行政执法体制改革，全面落实行政执法责任制，完善执法程序，细化、量化行政裁量标准，加强综合执法，着力解决权责交叉、多头执法以及不规范、不严格、不透明、不文明等问题。

提升依法行政水平。把法治思维、法治方式贯穿于政府工作全过程，坚持法定职责必须为、法无授权不可为，进一步强化落实之责，促进坚持依法行政、提高行政效能、提升服务水平三者结合。认真落实政务信息公开主体责任，以公开为常态、不公开为例外为原则，实现行政行为从决策到结果全过程公开透明。认真执行市人大及其常委会的决议、决定，依法报告工作，自觉接受监督。积极支持人民政协履行政治协商、民主监督、参政议政职能。认真办理人大代表建议和政协委员提案。支持各民主党派、工商联、无党派人士和工会、共青团、妇联等人民团体开展工作。

突显严实清廉高效。一是切实加强作风建设。扎实开展“三严三实”和“忠诚、干净、担当”专题教育，着力推进重点领域专项整治，切实增强党员领导干部践行“三严三实”的自觉性，锻造对党忠诚的政治品格，坚守个人干净的为官底线，奉行敢于担当的从政准则。巩固和拓展党的群众路线教育实践活动成果，严守“八项规定”，坚决反对“四风”，抓好市政府整改方案74条措施的落实，采取有效办法，把转变作风、改进方法与破解难题、推进发展有机结合起来，确保以整改实效取信于民。强化制度建设和正风肃纪，切实转变文风会风，让良好作风成为新常态。二是严格推行廉洁从政。全面推进惩治和预防腐败制度建设，围绕权力运行扎紧制度笼子，重点加强行政审批、招投标、公共资源交易、财政资金及国有资产等领域监管，促进监管工作规范化、法治化。继续加强政风行风建设，切实纠正不正之风。严控“三公”经费和会议经费，严格执行公务接待、公务出差、下基层交纳伙食费等制度规定。强化行政监察和审计监督，严肃查处违纪违法行为，坚决整治为官不为，坚决克服庸懒散软，坚决惩处失职渎职。认真落实党风廉政建设责任制，管好班子，以上率下，干净干事，清正廉洁。三是强化督查落实。全面落实工作责任，“一把手”带头抓落实、抓重点、抓督查，健全目标考核制度，推行重点项目、重点工作联动考核，强化督查针对性和实效性，敢于亮丑揭短，确保政令畅通，工作有效落实，着力提高政府执行力。

各位代表！一元复始，播下希望种子；万众一心，坚定发展信念！回首2014，我们同向同行克服了诸多困难，实现了稳中求进、和谐发展；放眼2015，我们同心同德去应对各种挑战，必将夺取更好更快更稳发展。山高自有人行路，水深不乏破浪舟。只要我们树立功成不必在我、成功必定有我的观念，坚定信心、胸怀壮志，取法乎上、狠抓落实，就没有翻不过的山，就没有迈不过的坎。让我们紧密团结在以习近平同志为核心的党中央周围，在省委、省政府和市委的坚强领导下，在市人大、市政协的严格监督和大力支持下，恪尽职守、锐意进取、开拓创新、奋力拼搏，聚精会神抓建设，一心一意推发展，全面完成“十二五”目标任务，为“十三五”全面协调可持续发展奠定更加坚实的基础。
